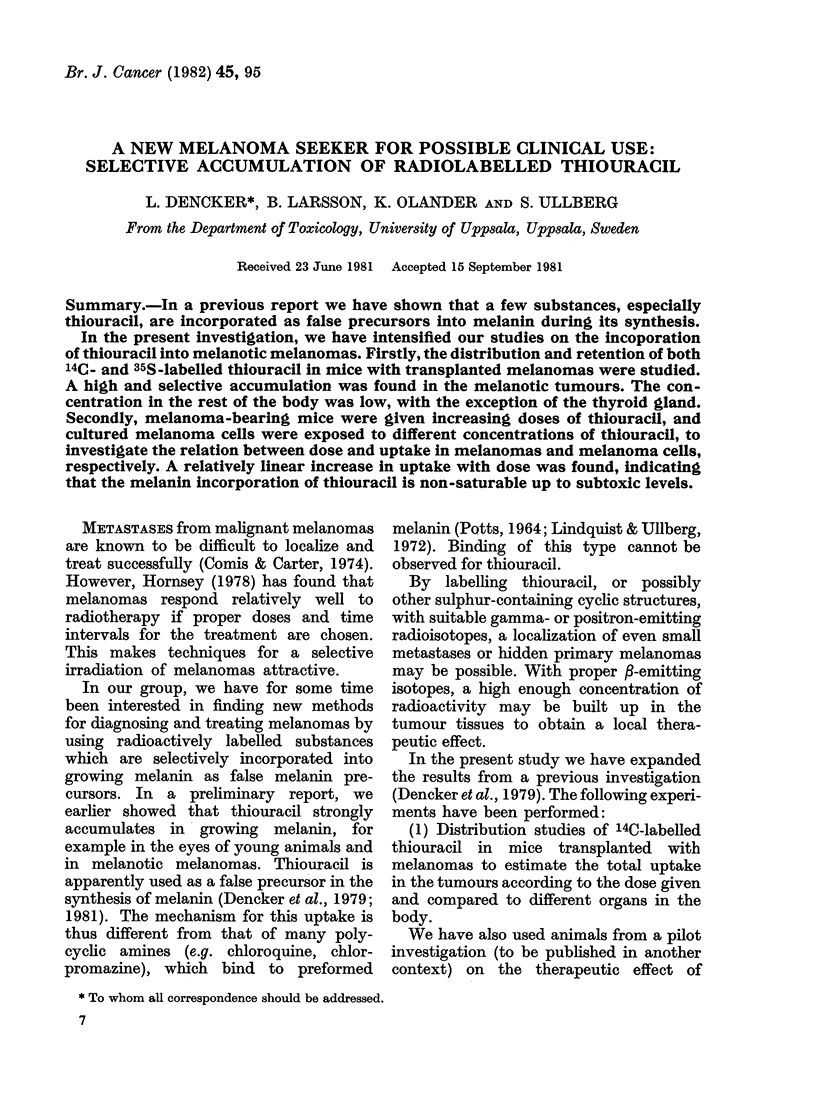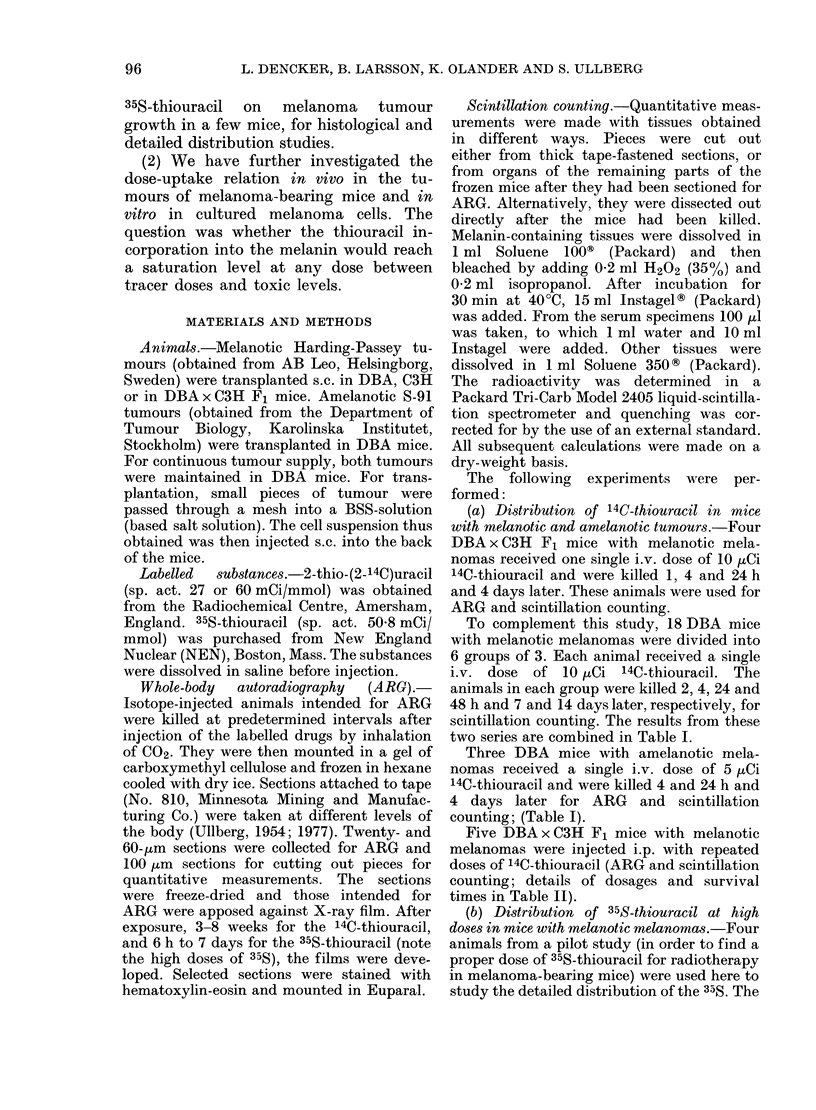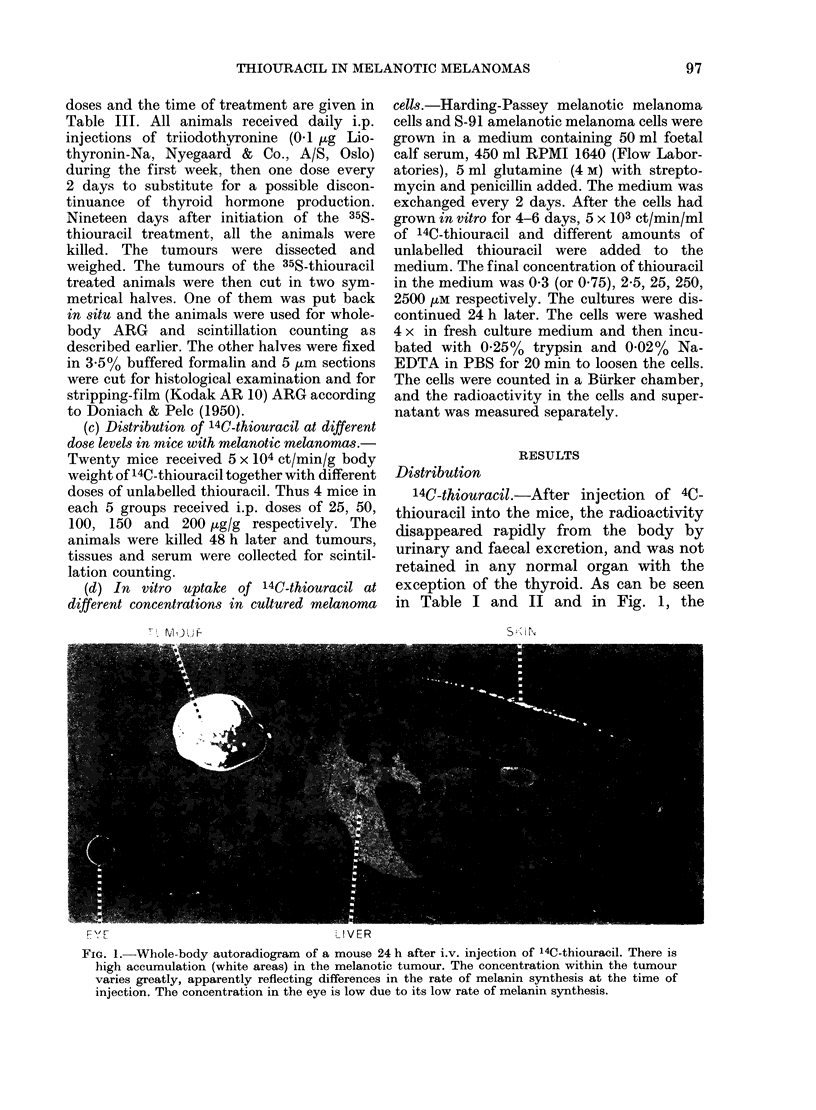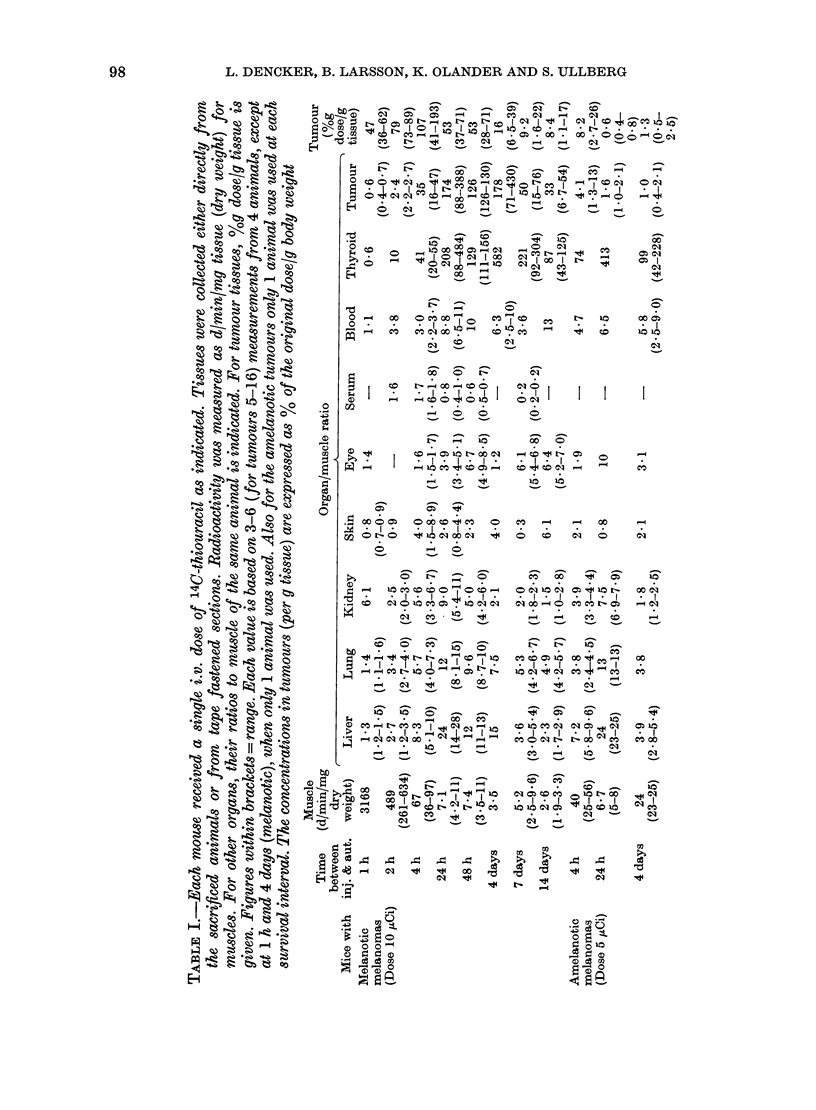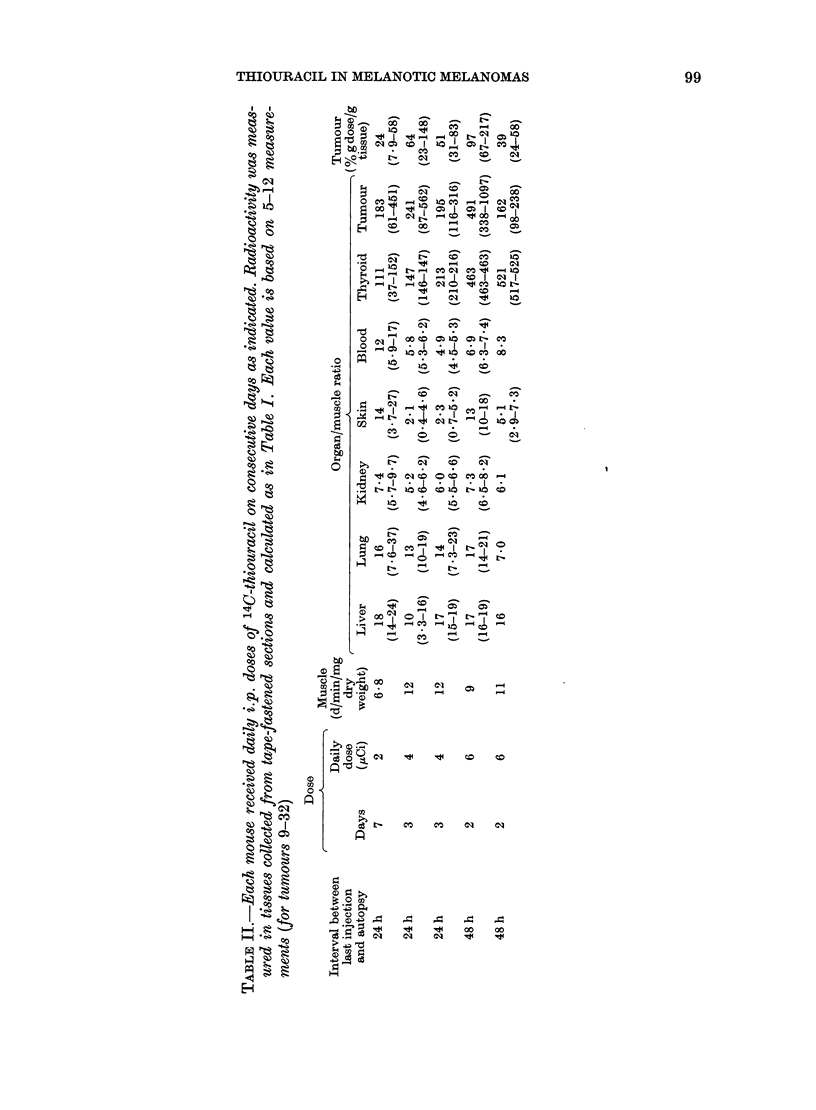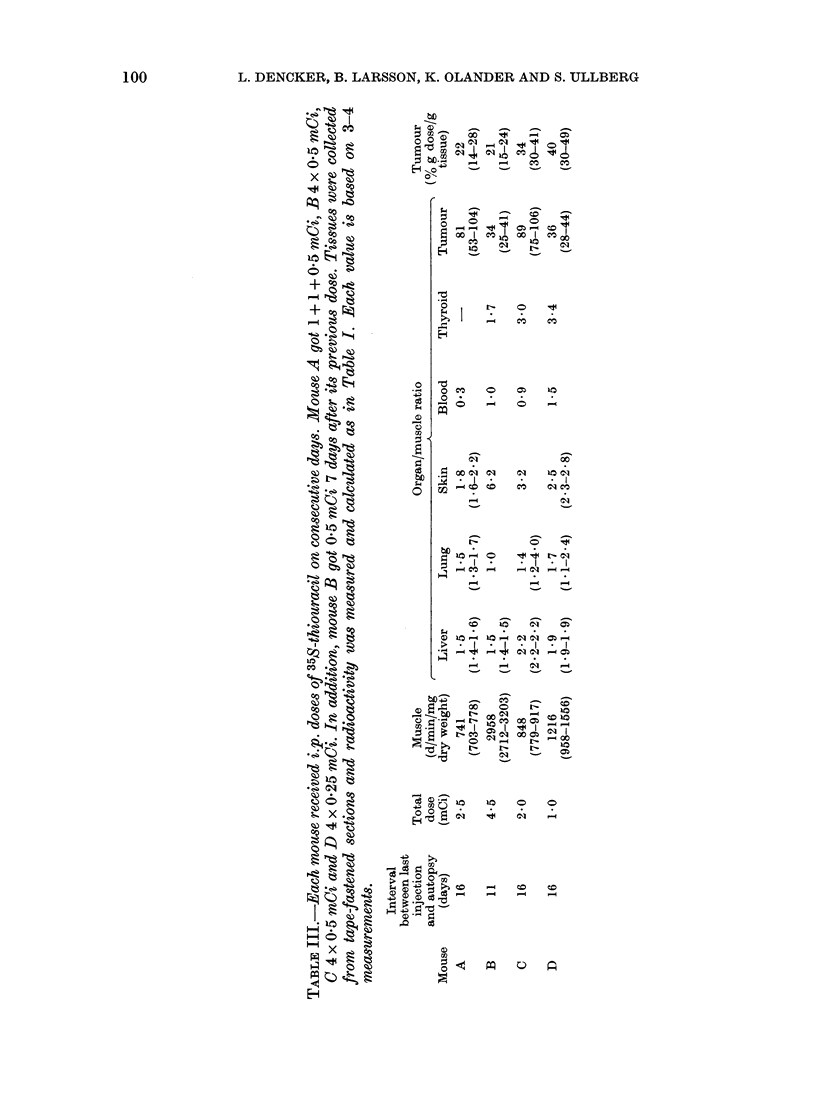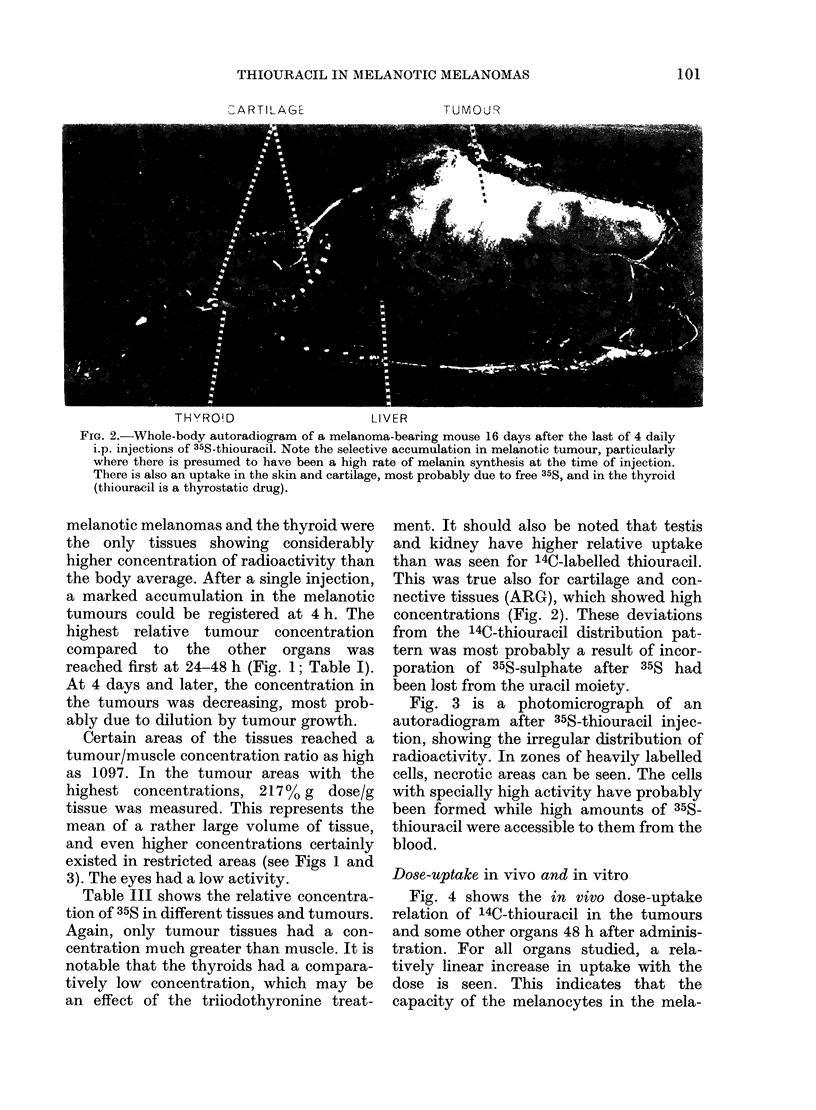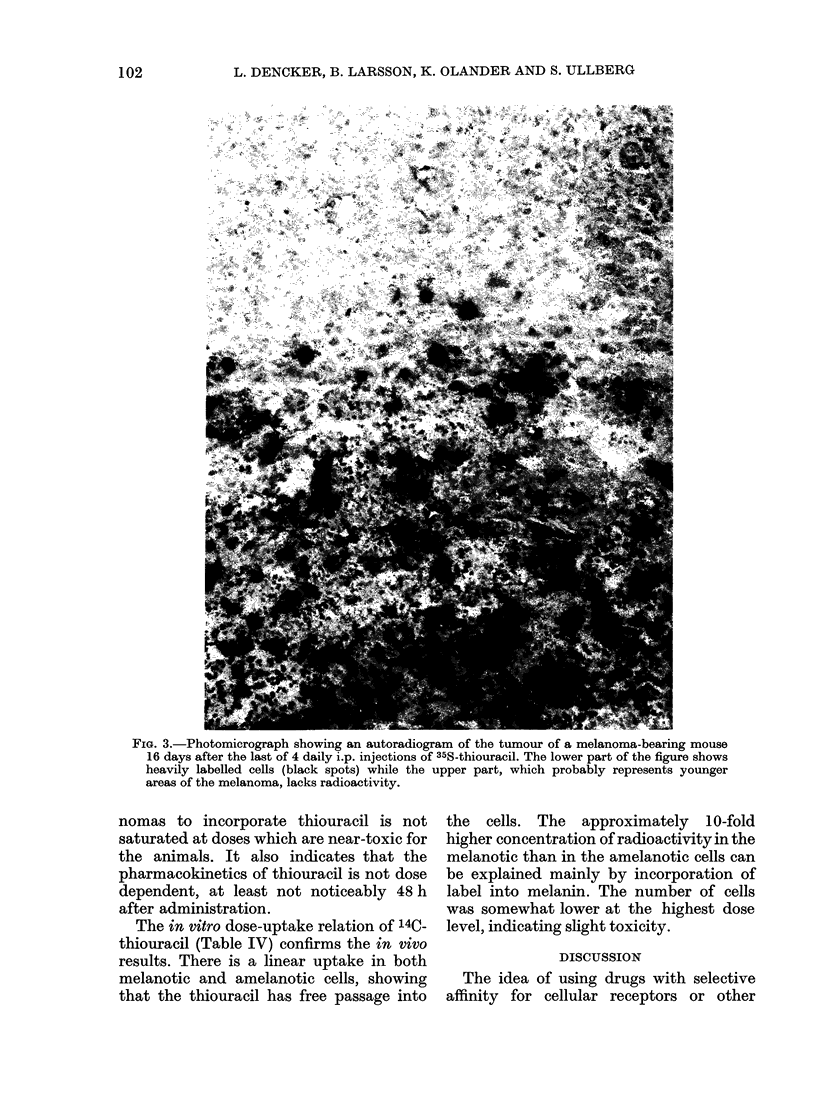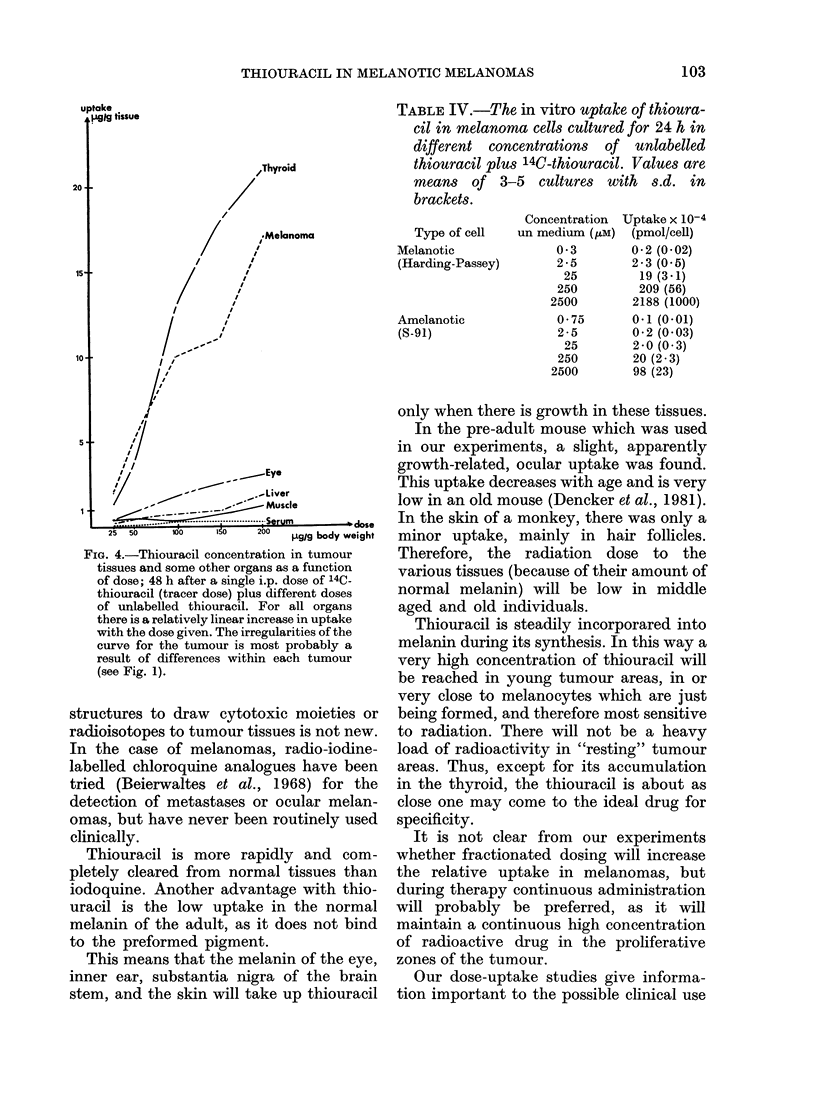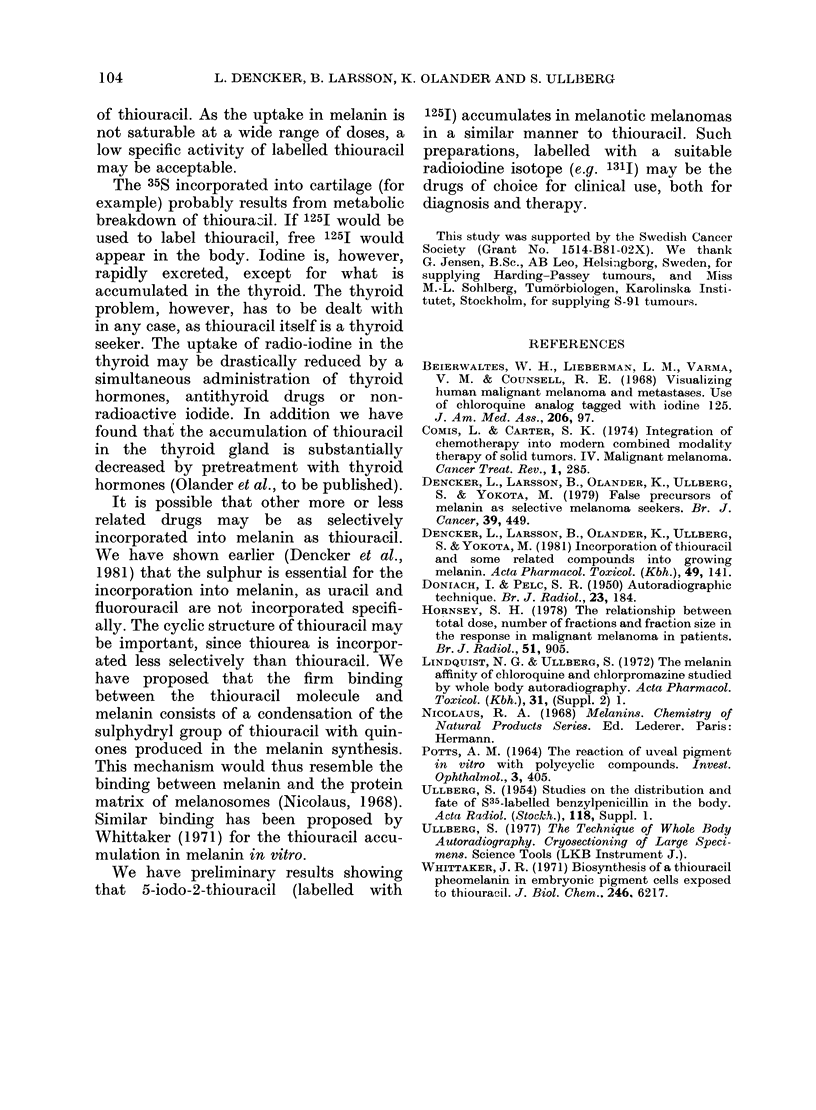# A new melanoma seeker for possible clinical use: selective accumulation of radiolabelled thiouracil.

**DOI:** 10.1038/bjc.1982.12

**Published:** 1982-01

**Authors:** L. Dencker, B. Larsson, K. Olander, S. Ullberg

## Abstract

**Images:**


Br. J. Cancer (1982) 45, 95

### A NEW MELANOMA SEEKER FOR POSSIBLE CLINICAL USE: SELECTIVE ACCUMULATION OF RADIOLABELLED THIOURACIL

L. DENCKER*, B. LARSSON, K. OLANDER AND S. ULLBERG

*From the Department of Toxicology, University of Uppsala, Uppsala, Sweden*

Received 23 June 1981 Accepted 15 September 1981

**Summary.—In a previous report we have shown that a few substances, especially thiouracil, are incorporated as false precursors into melanin during its synthesis.**

**In the present investigation, we have intensified our studies on the incoporation of thiouracil into melanotic melanomas. Firstly, the distribution and retention of both $^{14}C$- and $^{35}S$-labelled thiouracil in mice with transplanted melanomas were studied. A high and selective accumulation was found in the melanotic tumours. The concentration in the rest of the body was low, with the exception of the thyroid gland. Secondly, melanoma-bearing mice were given increasing doses of thiouracil, and cultured melanoma cells were exposed to different concentrations of thiouracil, to investigate the relation between dose and uptake in melanomas and melanoma cells, respectively. A relatively linear increase in uptake with dose was found, indicating that the melanin incorporation of thiouracil is non-saturable up to subtoxic levels.**

METASTASES from malignant melanomas are known to be difficult to localize and treat successfully (Comis & Carter, 1974). However, Hornsey (1978) has found that melanomas respond relatively well to radiotherapy if proper doses and time intervals for the treatment are chosen. This makes techniques for a selective irradiation of melanomas attractive.

In our group, we have for some time been interested in finding new methods for diagnosing and treating melanomas by using radioactively labelled substances which are selectively incorporated into growing melanin as false melanin precursors. In a preliminary report, we earlier showed that thiouracil strongly accumulates in growing melanin, for example in the eyes of young animals and in melanotic melanomas. Thiouracil is apparently used as a false precursor in the synthesis of melanin (Dencker *et al.*, 1979; 1981). The mechanism for this uptake is thus different from that of many polycyclic amines (*e.g.* chloroquine, chlorpromazine), which bind to preformed melanin (Potts, 1964; Lindquist & Ullberg, 1972). Binding of this type cannot be observed for thiouracil.

By labelling thiouracil, or possibly other sulphur-containing cyclic structures, with suitable gamma- or positron-emitting radioisotopes, a localization of even small metastases or hidden primary melanomas may be possible. With proper $\beta$-emitting isotopes, a high enough concentration of radioactivity may be built up in the tumour tissues to obtain a local therapeutic effect.

In the present study we have expanded the results from a previous investigation (Dencker *et al.*, 1979). The following experiments have been performed:

(1) Distribution studies of $^{14}C$-labelled thiouracil in mice transplanted with melanomas to estimate the total uptake in the tumours according to the dose given and compared to different organs in the body.

We have also used animals from a pilot investigation (to be published in another context) on the therapeutic effect of

* To whom all correspondence should be addressed.

$^{35}$S-thiouracil on melanoma tumour growth in a few mice, for histological and detailed distribution studies.

(2) We have further investigated the dose-uptake relation *in vivo* in the tumours of melanoma-bearing mice and *in vitro* in cultured melanoma cells. The question was whether the thiouracil incorporation into the melanin would reach a saturation level at any dose between tracer doses and toxic levels.

### MATERIALS AND METHODS

*Animals.*—Melanotic Harding-Passey tumours (obtained from AB Leo, Helsingborg, Sweden) were transplanted s.c. in DBA, C3H or in DBA × C3H $F_1$ mice. Amelanotic S-91 tumours (obtained from the Department of Tumour Biology, Karolinska Institutet, Stockholm) were transplanted in DBA mice. For continuous tumour supply, both tumours were maintained in DBA mice. For transplantation, small pieces of tumour were passed through a mesh into a BSS-solution (based salt solution). The cell suspension thus obtained was then injected s.c. into the back of the mice.

*Labelled substances.*—2-thio-(2-$^{14}$C)uracil (sp. act. 27 or 60 mCi/mmol) was obtained from the Radiochemical Centre, Amersham, England. $^{35}$S-thiouracil (sp. act. 50·8 mCi/mmol) was purchased from New England Nuclear (NEN), Boston, Mass. The substances were dissolved in saline before injection.

*Whole-body autoradiography (ARG).*—Isotope-injected animals intended for ARG were killed at predetermined intervals after injection of the labelled drugs by inhalation of $CO_2$. They were then mounted in a gel of carboxymethyl cellulose and frozen in hexane cooled with dry ice. Sections attached to tape (No. 810, Minnesota Mining and Manufacturing Co.) were taken at different levels of the body (Ullberg, 1954; 1977). Twenty- and 60-$\mu$m sections were collected for ARG and 100 $\mu$m sections for cutting out pieces for quantitative measurements. The sections were freeze-dried and those intended for ARG were apposed against X-ray film. After exposure, 3–8 weeks for the $^{14}$C-thiouracil, and 6 h to 7 days for the $^{35}$S-thiouracil (note the high doses of $^{35}$S), the films were developed. Selected sections were stained with hematoxylin-eosin and mounted in Euparal.

*Scintillation counting.*—Quantitative measurements were made with tissues obtained in different ways. Pieces were cut out either from thick tape-fastened sections, or from organs of the remaining parts of the frozen mice after they had been sectioned for ARG. Alternatively, they were dissected out directly after the mice had been killed. Melanin-containing tissues were dissolved in 1 ml Soluene 100® (Packard) and then bleached by adding 0·2 ml $H_2O_2$ (35%) and 0·2 ml isopropanol. After incubation for 30 min at 40°C, 15 ml Instagel® (Packard) was added. From the serum specimens 100 $\mu$l was taken, to which 1 ml water and 10 ml Instagel were added. Other tissues were dissolved in 1 ml Soluene 350® (Packard). The radioactivity was determined in a Packard Tri-Carb Model 2405 liquid-scintillation spectrometer and quenching was corrected for by the use of an external standard. All subsequent calculations were made on a dry-weight basis.

The following experiments were performed:

(*a*) *Distribution of $^{14}$C-thiouracil in mice with melanotic and amelanotic tumours.*—Four DBA × C3H $F_1$ mice with melanotic melanomas received one single i.v. dose of 10 $\mu$Ci $^{14}$C-thiouracil and were killed 1, 4 and 24 h and 4 days later. These animals were used for ARG and scintillation counting.

To complement this study, 18 DBA mice with melanotic melanomas were divided into 6 groups of 3. Each animal received a single i.v. dose of 10 $\mu$Ci $^{14}$C-thiouracil. The animals in each group were killed 2, 4, 24 and 48 h and 7 and 14 days later, respectively, for scintillation counting. The results from these two series are combined in Table I.

Three DBA mice with amelanotic melanomas received a single i.v. dose of 5 $\mu$Ci $^{14}$C-thiouracil and were killed 4 and 24 h and 4 days later for ARG and scintillation counting; (Table I).

Five DBA × C3H $F_1$ mice with melanotic melanomas were injected i.p. with repeated doses of $^{14}$C-thiouracil (ARG and scintillation counting; details of dosages and survival times in Table II).

(*b*) *Distribution of $^{35}$S-thiouracil at high doses in mice with melanotic melanomas.*—Four animals from a pilot study (in order to find a proper dose of $^{35}$S-thiouracil for radiotherapy in melanoma-bearing mice) were used here to study the detailed distribution of the $^{35}$S. The doses and the time of treatment are given in Table III. All animals received daily i.p. injections of triiodothyronine (0·1 μg Liothyronin-Na, Nyegaard & Co., A/S, Oslo) during the first week, then one dose every 2 days to substitute for a possible discontinuance of thyroid hormone production. Nineteen days after initiation of the $^{35}$S-thiouracil treatment, all the animals were killed. The tumours were dissected and weighed. The tumours of the $^{35}$S-thiouracil treated animals were then cut in two symmetrical halves. One of them was put back *in situ* and the animals were used for whole-body ARG and scintillation counting as described earlier. The other halves were fixed in 3·5% buffered formalin and 5 μm sections were cut for histological examination and for stripping-film (Kodak AR 10) ARG according to Doniach & Pelc (1950).

(*c*) *Distribution of $^{14}$C-thiouracil at different dose levels in mice with melanotic melanomas.*—Twenty mice received $5 \times 10^4$ ct/min/g body weight of $^{14}$C-thiouracil together with different doses of unlabelled thiouracil. Thus 4 mice in each 5 groups received i.p. doses of 25, 50, 100, 150 and 200 μg/g respectively. The animals were killed 48 h later and tumours, tissues and serum were collected for scintillation counting.

(*d*) *In vitro uptake of $^{14}$C-thiouracil at different concentrations in cultured melanoma cells.*—Harding-Passey melanotic melanoma cells and S-91 amelanotic melanoma cells were grown in a medium containing 50 ml foetal calf serum, 450 ml RPMI 1640 (Flow Laboratories), 5 ml glutamine (4 M) with streptomycin and penicillin added. The medium was exchanged every 2 days. After the cells had grown *in vitro* for 4–6 days, $5 \times 10^3$ ct/min/ml of $^{14}$C-thiouracil and different amounts of unlabelled thiouracil were added to the medium. The final concentration of thiouracil in the medium was 0·3 (or 0·75), 2·5, 25, 250, 2500 μM respectively. The cultures were discontinued 24 h later. The cells were washed 4 × in fresh culture medium and then incubated with 0·25% trypsin and 0·02% Na-EDTA in PBS for 20 min to loosen the cells. The cells were counted in a Bürker chamber, and the radioactivity in the cells and supernatant was measured separately.

### RESULTS

### *Distribution*

*$^{14}$C-thiouracil.*—After injection of $^4$C-thiouracil into the mice, the radioactivity disappeared rapidly from the body by urinary and faecal excretion, and was not retained in any normal organ with the exception of the thyroid. As can be seen in Table I and II and in Fig. 1, the

FIG. 1.—Whole-body autoradiogram of a mouse 24 h after i.v. injection of $^{14}$C-thiouracil. There is high accumulation (white areas) in the melanotic tumour. The concentration within the tumour varies greatly, apparently reflecting differences in the rate of melanin synthesis at the time of injection. The concentration in the eye is low due to its low rate of melanin synthesis.

FIG. 2.—Whole-body autoradiogram of a melanoma-bearing mouse 16 days after the last of 4 daily i.p. injections of $^{35}$S-thiouracil. Note the selective accumulation in melanotic tumour, particularly where there is presumed to have been a high rate of melanin synthesis at the time of injection. There is also an uptake in the skin and cartilage, most probably due to free $^{35}$S, and in the thyroid (thiouracil is a thyrostatic drug).

melanotic melanomas and the thyroid were the only tissues showing considerably higher concentration of radioactivity than the body average. After a single injection, a marked accumulation in the melanotic tumours could be registered at 4 h. The highest relative tumour concentration compared to the other organs was reached first at 24–48 h (Fig. 1; Table I). At 4 days and later, the concentration in the tumours was decreasing, most probably due to dilution by tumour growth.

Certain areas of the tissues reached a tumour/muscle concentration ratio as high as 1097. In the tumour areas with the highest concentrations, 217% g dose/g tissue was measured. This represents the mean of a rather large volume of tissue, and even higher concentrations certainly existed in restricted areas (see Figs 1 and 3). The eyes had a low activity.

Table III shows the relative concentration of $^{35}$S in different tissues and tumours. Again, only tumour tissues had a concentration much greater than muscle. It is notable that the thyroids had a comparatively low concentration, which may be an effect of the triiodothyronine treatment. It should also be noted that testis and kidney have higher relative uptake than was seen for $^{14}$C-labelled thiouracil. This was true also for cartilage and connective tissues (ARG), which showed high concentrations (Fig. 2). These deviations from the $^{14}$C-thiouracil distribution pattern was most probably a result of incorporation of $^{35}$S-sulphate after $^{35}$S had been lost from the uracil moiety.

Fig. 3 is a photomicrograph of an autoradiogram after $^{35}$S-thiouracil injection, showing the irregular distribution of radioactivity. In zones of heavily labelled cells, necrotic areas can be seen. The cells with specially high activity have probably been formed while high amounts of $^{35}$S-thiouracil were accessible to them from the blood.

### *Dose-uptake* in vivo *and* in vitro

Fig. 4 shows the *in vivo* dose-uptake relation of $^{14}$C-thiouracil in the tumours and some other organs 48 h after administration. For all organs studied, a relatively linear increase in uptake with the dose is seen. This indicates that the capacity of the melanocytes in the mela-

FIG. 3.—Photomicrograph showing an autoradiogram of the tumour of a melanoma-bearing mouse 16 days after the last of 4 daily i.p. injections of $^{35}$S-thiouracil. The lower part of the figure shows heavily labelled cells (black spots) while the upper part, which probably represents younger areas of the melanoma, lacks radioactivity.

nomas to incorporate thiouracil is not saturated at doses which are near-toxic for the animals. It also indicates that the pharmacokinetics of thiouracil is not dose dependent, at least not noticeably 48 h after administration.

The *in vitro* dose-uptake relation of $^{14}$C-thiouracil (Table IV) confirms the *in vivo* results. There is a linear uptake in both melanotic and amelanotic cells, showing that the thiouracil has free passage into the cells. The approximately 10-fold higher concentration of radioactivity in the melanotic than in the amelanotic cells can be explained mainly by incorporation of label into melanin. The number of cells was somewhat lower at the highest dose level, indicating slight toxicity.

### DISCUSSION

The idea of using drugs with selective affinity for cellular receptors or other

FIG. 4.—Thiouracil concentration in tumour tissues and some other organs as a function of dose; 48 h after a single i.p. dose of $^{14}$C-thiouracil (tracer dose) plus different doses of unlabelled thiouracil. For all organs there is a relatively linear increase in uptake with the dose given. The irregularities of the curve for the tumour is most probably a result of differences within each tumour (see Fig. 1).

structures to draw cytotoxic moieties or radioisotopes to tumour tissues is not new. In the case of melanomas, radio-iodine-labelled chloroquine analogues have been tried (Beierwaltes *et al.*, 1968) for the detection of metastases or ocular melanomas, but have never been routinely used clinically.

Thiouracil is more rapidly and completely cleared from normal tissues than iodoquine. Another advantage with thiouracil is the low uptake in the normal melanin of the adult, as it does not bind to the preformed pigment.

This means that the melanin of the eye, inner ear, substantia nigra of the brain stem, and the skin will take up thiouracil only when there is growth in these tissues.

TABLE IV.—*The* in vitro *uptake of thiouracil in melanoma cells cultured for 24 h in different concentrations of unlabelled thiouracil plus $^{14}$C-thiouracil. Values are means of 3–5 cultures with s.d. in brackets.*

| Type of cell | Concentration un medium (µM) | Uptake × $10^{-4}$ (pmol/cell) |
|---|---|---|
| Melanotic | 0·3 | 0·2 (0·02) |
| (Harding-Passey) | 2·5 | 2·3 (0·5) |
| | 25 | 19 (3·1) |
| | 250 | 209 (56) |
| | 2500 | 2188 (1000) |
| Amelanotic | 0·75 | 0·1 (0·01) |
| (S-91) | 2·5 | 0·2 (0·03) |
| | 25 | 2·0 (0·3) |
| | 250 | 20 (2·3) |
| | 2500 | 98 (23) |

In the pre-adult mouse which was used in our experiments, a slight, apparently growth-related, ocular uptake was found. This uptake decreases with age and is very low in an old mouse (Dencker *et al.*, 1981). In the skin of a monkey, there was only a minor uptake, mainly in hair follicles. Therefore, the radiation dose to the various tissues (because of their amount of normal melanin) will be low in middle aged and old individuals.

Thiouracil is steadily incorporared into melanin during its synthesis. In this way a very high concentration of thiouracil will be reached in young tumour areas, in or very close to melanocytes which are just being formed, and therefore most sensitive to radiation. There will not be a heavy load of radioactivity in "resting" tumour areas. Thus, except for its accumulation in the thyroid, the thiouracil is about as close one may come to the ideal drug for specificity.

It is not clear from our experiments whether fractionated dosing will increase the relative uptake in melanomas, but during therapy continuous administration will probably be preferred, as it will maintain a continuous high concentration of radioactive drug in the proliferative zones of the tumour.

Our dose-uptake studies give information important to the possible clinical use of thiouracil. As the uptake in melanin is not saturable at a wide range of doses, a low specific activity of labelled thiouracil may be acceptable.

The $^{35}S$ incorporated into cartilage (for example) probably results from metabolic breakdown of thiouracil. If $^{125}I$ would be used to label thiouracil, free $^{125}I$ would appear in the body. Iodine is, however, rapidly excreted, except for what is accumulated in the thyroid. The thyroid problem, however, has to be dealt with in any case, as thiouracil itself is a thyroid seeker. The uptake of radio-iodine in the thyroid may be drastically reduced by a simultaneous administration of thyroid hormones, antithyroid drugs or non-radioactive iodide. In addition we have found that the accumulation of thiouracil in the thyroid gland is substantially decreased by pretreatment with thyroid hormones (Olander *et al.*, to be published).

It is possible that other more or less related drugs may be as selectively incorporated into melanin as thiouracil. We have shown earlier (Dencker *et al.*, 1981) that the sulphur is essential for the incorporation into melanin, as uracil and fluorouracil are not incorporated specifically. The cyclic structure of thiouracil may be important, since thiourea is incorporated less selectively than thiouracil. We have proposed that the firm binding between the thiouracil molecule and melanin consists of a condensation of the sulphydryl group of thiouracil with quinones produced in the melanin synthesis. This mechanism would thus resemble the binding between melanin and the protein matrix of melanosomes (Nicolaus, 1968). Similar binding has been proposed by Whittaker (1971) for the thiouracil accumulation in melanin *in vitro*.

We have preliminary results showing that 5-iodo-2-thiouracil (labelled with $^{125}I$) accumulates in melanotic melanomas in a similar manner to thiouracil. Such preparations, labelled with a suitable radioiodine isotope (*e.g.* $^{131}I$) may be the drugs of choice for clinical use, both for diagnosis and therapy.

This study was supported by the Swedish Cancer Society (Grant No. 1514-B81-02X). We thank G. Jensen, B.Sc., AB Leo, Helsingborg, Sweden, for supplying Harding-Passey tumours, and Miss M.-L. Sohlberg, Tumörbiologen, Karolinska Institutet, Stockholm, for supplying S-91 tumours.

### REFERENCES

BEIERWALTES, W. H., LIEBERMAN, L. M., VARMA, V. M. & COUNSELL, R. E. (1968) Visualizing human malignant melanoma and metastases. Use of chloroquine analog tagged with iodine 125. *J. Am. Med. Ass.*, **206**, 97.

COMIS, L. & CARTER, S. K. (1974) Integration of chemotherapy into modern combined modality therapy of solid tumors. IV. Malignant melanoma. *Cancer Treat. Rev.*, **1**, 285.

DENCKER, L., LARSSON, B., OLANDER, K., ULLBERG, S. & YOKOTA, M. (1979) False precursors of melanin as selective melanoma seekers. *Br. J. Cancer*, **39**, 449.

DENCKER, L., LARSSON, B., OLANDER, K., ULLBERG, S. & YOKOTA, M. (1981) Incorporation of thiouracil and some related compounds into growing melanin. *Acta Pharmacol. Toxicol.* (*Kbh.*), **49**, 141.

DONIACH, I. & PELC, S. R. (1950) Autoradiographic technique. *Br. J. Radiol.*, **23**, 184.

HORNSEY, S. H. (1978) The relationship between total dose, number of fractions and fraction size in the response in malignant melanoma in patients. *Br. J. Radiol.*, **51**, 905.

LINDQUIST, N. G. & ULLBERG, S. (1972) The melanin affinity of chloroquine and chlorpromazine studied by whole body autoradiography. *Acta Pharmacol. Toxicol.* (*Kbh.*), **31**, (Suppl. 2) 1.

NICOLAUS, R. A. (1968) *Melanins. Chemistry of Natural Products Series.* Ed. Lederer. Paris: Hermann.

POTTS, A. M. (1964) The reaction of uveal pigment *in vitro* with polycyclic compounds. *Invest. Ophthalmol.*, **3**, 405.

ULLBERG, S. (1954) Studies on the distribution and fate of $S^{35}$-labelled benzylpenicillin in the body. *Acta Radiol.* (*Stockh.*), **118**, Suppl. 1.

ULLBERG, S. (1977) *The Technique of Whole Body Autoradiography. Cryosectioning of Large Specimens.* Science Tools (LKB Instrument J.).

WHITTAKER, J. R. (1971) Biosynthesis of a thiouracil pheomelanin in embryonic pigment cells exposed to thiouracil. *J. Biol. Chem.*, **246**, 6217.